# Prevalence and patterns of traumatic dental injuries in primary teeth: a 3-year retrospective overview study in Vienna

**DOI:** 10.1007/s00784-021-04190-2

**Published:** 2021-11-06

**Authors:** Sophie Lembacher, Steffen Schneider, Steffen Lettner, Katrin Bekes

**Affiliations:** 1 Medical University of Vienna, University Clinic of Dentistry, Department of Paediatric Dentistry, Sensengasse 2a, Vienna, 1090 Austria; 2 Medical University of Vienna, Department of Oral and Maxillofacial Surgery, Spitalgasse 23, Vienna, 1090 Austria; 3 Medical University of Vienna, Karl Donath Laboratory for Hard Tissue and Biomaterial Research, Statistics, School of Dentistry, Sensengasse 2a, Vienna, 1090 Austria

**Keywords:** Traumatic dental injuries, Primary dentition, Incidence, Tooth fracture, Dislocation, Austria

## Abstract

**Objectives:**

The aim of this study was to retrospectively identify the prevalence, patterns, and accident types of traumatic dental injuries (TDIs) in children with primary teeth in Vienna, Austria.

**Material and methods:**

The investigation was conducted as a retrospective overview study including all children with TDIs in primary teeth at the University Dental Clinic of Vienna (Austria) between 2014 and 2016. Dental records including age, gender, location of trauma, type of trauma, cause of TDI, and location of traumatic incident were obtained. Furthermore, the time of presentation and the time span between TDI and initial treatment were evaluated.

**Results:**

The sample comprised TDIs in 243 patients with 403 primary teeth. In a ratio of 1:1.45, boys were significantly more involved than girls. Upper central and lateral incisors were most frequently affected (*n* = 371, 92.1%). Dislocations were the most common type of injury (*n* = 298, 74%) with subluxations being the most prevalent form (*n* = 85, 28.5%). In 23% (*n* = 92), fractures were observed. The majority of traumatic incidents occurred at home (88.5%).

**Conclusion:**

The injury characteristics are comparable to what has previously been reported in other studies in pediatric populations.

**Clinical relevance:**

TDIs are a prevalent event in children worldwide and incisors are the most affected teeth in the primary dentition. Thus, dental practitioners should be able to handle these injuries.

## Introduction

Traumatic dental injuries (TDIs) are one of the most serious dental public health problems among the younger population as most injuries occur in childhood or adolescence. For children aged 0 to 6 years, oral injuries account for as much as 17% of all bodily injuries [1]. Approximately one-third of toddlers and preschool children suffer dental trauma involving the primary dentition. In many cases, a dental traumatic injury in deciduous teeth is the reason for the child’s first visit to the dentist [2, 3]. A structured approach to minimize anxiety and provide immediate care is essential and poses a challenge to parents as well as clinicians in pediatric dentistry. But TDIs do not only cause painful and distressing events. They also may have aesthetic, functional, economic, and psychosocial effects [4–6]. In addition to immediately measurable clinical consequences, potential sequelae to developing succedaneous permanent teeth including hypoplasia, root dilacerations, and other enamel or development disturbances present lasting complications [7–9]. Furthermore, TDIs in deciduous teeth increase the risk for dental trauma in the permanent dentition [10]. This highlights the impact that TDIs in primary teeth have on public health. The prognosis of traumatized teeth is equally dependent on the quality of immediate emergency care at the time and place of traumatic incident and initial professional medical assessment and treatment [1, 11]. Several publications have discussed dental trauma in the primary and permanent dentition. The global prevalence of TDIs in primary teeth ranges between 11 and 47% [1, 2, 12]. The International Association of Dental Traumatology estimates the prevalence at 22.7% [3, 13]. The considerable variability in reported prevalence of TDIs likely derives from incoherence in applied trauma classification systems, geographical, socioeconomic, and behavioral differences between study locations and countries [14]. Considering that many apparently minor injuries remain unreported, the number of TDIs is likely to be underestimated [2]. The Department of Emergency Dental Care at the University Dental Clinic in Vienna is the only specialized unit for TDIs in the area within the public university system. In view of 944 local dentist offices, two additional hospitals with dental-care units, and 18 outpatient clinics, the tendency for a decentralized approach in regard to provision for dental care in Vienna, a city with 1.87 million inhabitants, is apparent.

However, so far there has only been little epidemiological research in dental traumatology in Austria. Existing epidemiologic data primarily focuses on dental trauma in the context of facial injuries or is based on selective patient cohorts limited to different injury or accident types in permanent teeth [15, 16]. Therefore, the aim of the present retrospective study was to evaluate TDIs in primary teeth in Vienna between 2014 and 2016. Several variables relating to TDIs were analyzed including gender, type of injury, number of affected teeth, location of traumatic incident, and causes of TDIs.

## Material and methods

The study was conducted as a retrospective overview study including all children with a TDI in the primary dentition who presented at the Department of Emergency Dental Care at the University Dental Clinic in Vienna (Austria) between January 2014 and December 2016. Medical records were retrieved from the electronic patient registry. The data provided was registered by 26 practitioners of varying backgrounds in regard to their specialization during their routine shifts at the Department of Emergency Dental Care. 64.2% (*n* = 156) of patients were registered by permanent residents of the Emergency Care Unit, 16% (*n* = 39) by pediatric dentists, and 19.8% (*n* = 48) by practitioners of varying other specializations (e.g., oral surgeons, endodontists, prosthodontists). All TDIs affecting primary teeth were considered for inclusion in the study. Exclusion criteria comprised injuries to permanent teeth or deficient records. Relevant data were classified and grouped according to age, gender, the time interval between trauma and arrival at the clinic, affected teeth, type of trauma, etiology of TDI, and location of traumatic incident. The classification of dental trauma was conducted in accordance with the classification system of the World Health Organization which was slightly modified by Andreasen et al. [8]. Hence, TDIs were classified into injuries to the dental hard tissues and pulp (enamel infraction, crown fracture without pulp exposure (uncomplicated crown fracture), crown fracture with pulp exposure (complicated crown fracture), crown-root fracture, root fracture) and injuries to the periodontal tissue (concussion, subluxation, lateral dislocation, intrusion, extrusion, and avulsion) (Table 1). Additionally, combined injuries were introduced as a third diagnostic entity which were defined as simultaneously occurring fractures and dislocations within the same tooth at the same time (meaning per trauma). Table 1 presents the three main categories of dental trauma and their subgroups observed in this study. Clinical oral and radiographic examinations were conducted in accordance with current guidelines of the German Society of Dental, Oral and Craniomandibular Sciences (DGZMK) [17]. Exceptions for radiographic diagnostics were only made if the patient was not compliant.

**Table 1:** Classification of dental trauma used in the present study [8]

Type of trauma	Abbreviation
Fracture Enamel infraction Crown fracture With pulp exposure No pulp exposure Crown-root fracture Root fracture Dislocation Concussion Subluxation Lateral dislocation Intrusion Extrusion Avulsion Combined injuries Fractures and dislocations simultaneously occurring within the same tooth at the same time	**F** EF CFP CFX CRF RF **D** CCS LOS LDL INT EXT AVU **C**

Statistical analysis was performed with R version 4.0.2 (R Core Team 2020, Austria). Descriptive statistics (mean, SD) were provided for continuous measurements (time to treatment, age). Nominal measurements (e.g., gender, type of injury, type of accident) are summarized using frequencies and proportions as well as crosstabulations. Wilson score intervals and *χ*^2^ tests with continuity correction were calculated to compare interesting proportions. For visualization kernel density estimators (KDE), a continuous version of histograms was used. Graphs were created using MS Excel™ 2016 (IBM, USA).

## Results

**Fig. 1:**
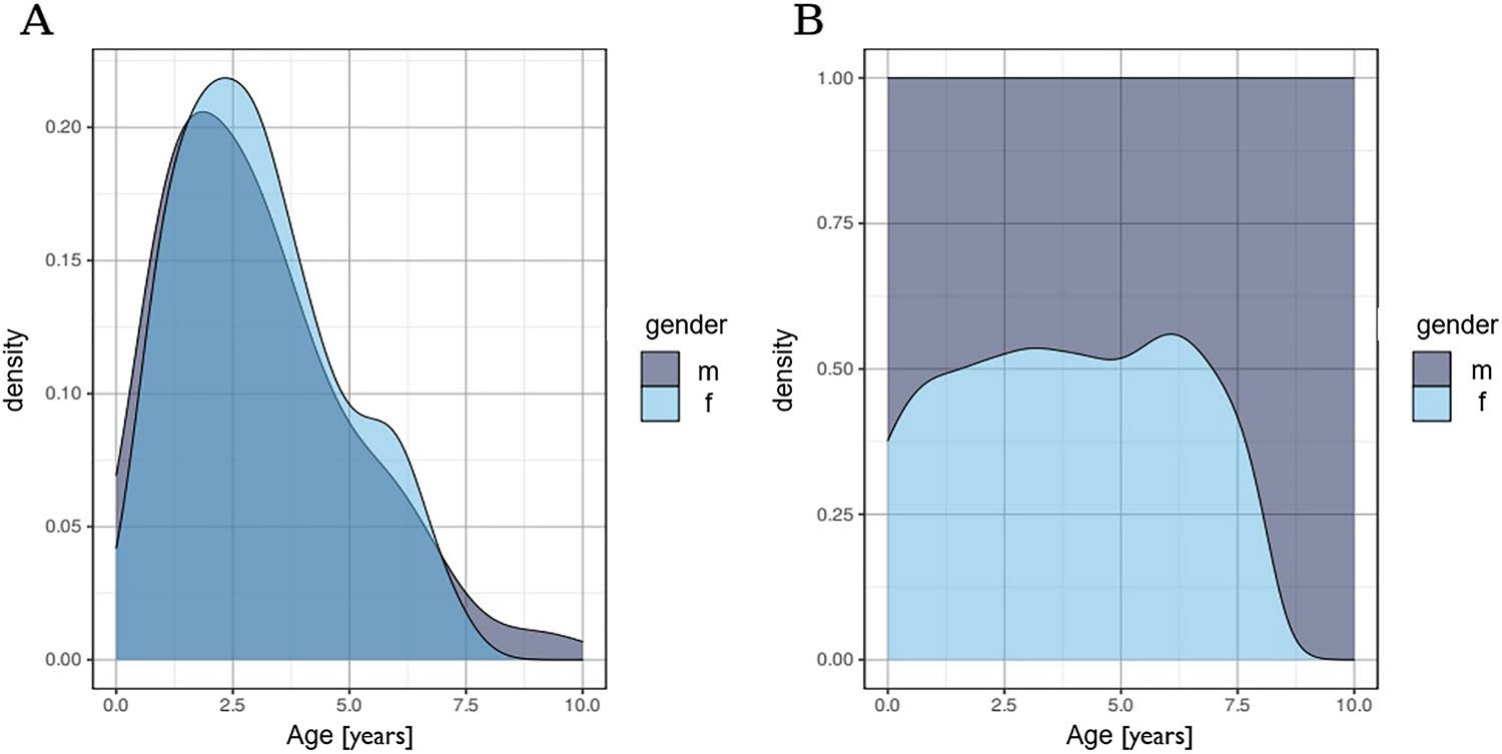
Age distribution of patients with regard to gender

The sample comprised 403 deciduous teeth of 243 patients with TDIs in the primary dentition. At the time of injury, the mean age was 3.08 years (± 1.9, range 0–10 years). A peak was observed between the first and second year of life (Fig. 1). With a gender ratio of 1:1.45, 59.3% (95% CI: 52.8–65.4%, *n* = 144) of the patients were males and 40.7% (95% CI: 34.6–47.2%, *n* = 99) females. With a *χ*^2^ test with Yates’ continuity correction calculating a *p*-value of 0.0048, boys were significantly more affected by TDIs than girls.

**Fig. 2:**
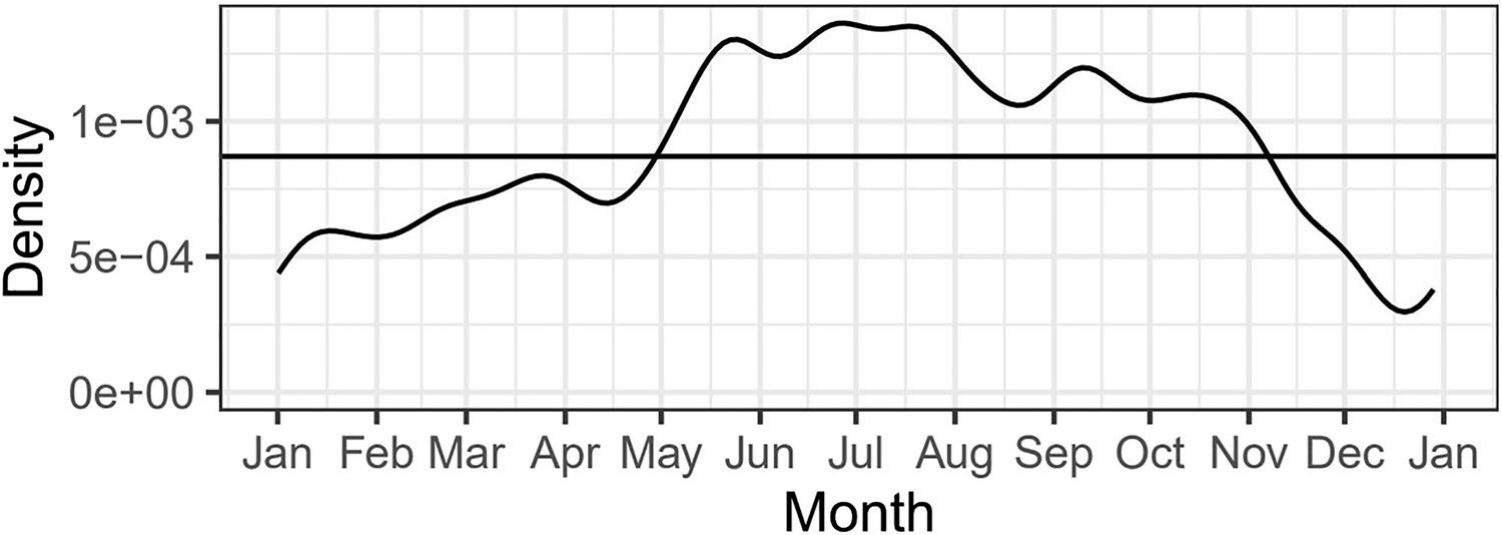
Kernel density estimation (KDE) plot for the relative likelihood of a TDI per date

**Fig. 3:**
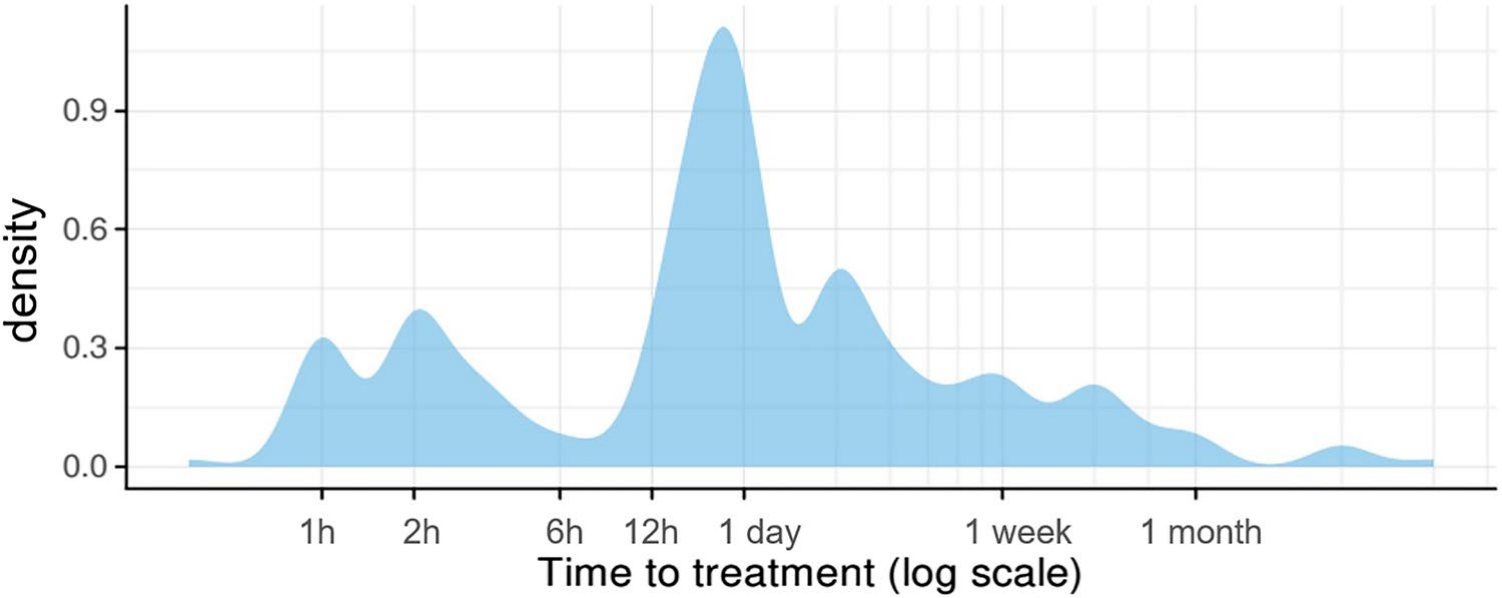
Time to treatment

In 77% (*n* = 187) of cases, patients (*n* = 187) immediately sought emergency care at the Department of Emergency Dental Care at the University Dental Clinic of Vienna, whereas 11.4% (*n* = 28) were referrals. One patient was referred by a general practitioner, one by a pediatrician and 10 patients by dentists. Sixteen patients sought immediate care after having been referred by emergency hospitals. For 28 patients, no information was available. The majority of patients presented during official clinic hours on weekdays, the busiest day being Friday (*n* = 41) (Table 2). Out of 220 accidents, 68 occurred on holidays or weekends. Figure 2 shows the relative likelihood of a TDI per date in a kernel density estimation (KDE) plot. The horizontal line shows average relative likelihood for comparison. From early November to the end of May, there were relatively fewer TDIs. A relatively higher amount of TDIs was observed from May to the beginning of November. With regard to clinic arrival time following dental trauma, 65.2% (*n* = 144) sought treatment within the first 24 h after TDI with a maximum at 2 h. Figure 3 shows the average time passed until treatment.

**Table 2:** Frequency of TDI occurrence per weekday

Day of the week	*n*
Monday Tuesday Wednesday Thursday Friday Saturday Sunday	31 26 24 34 41 30 34

**Fig. 4:**
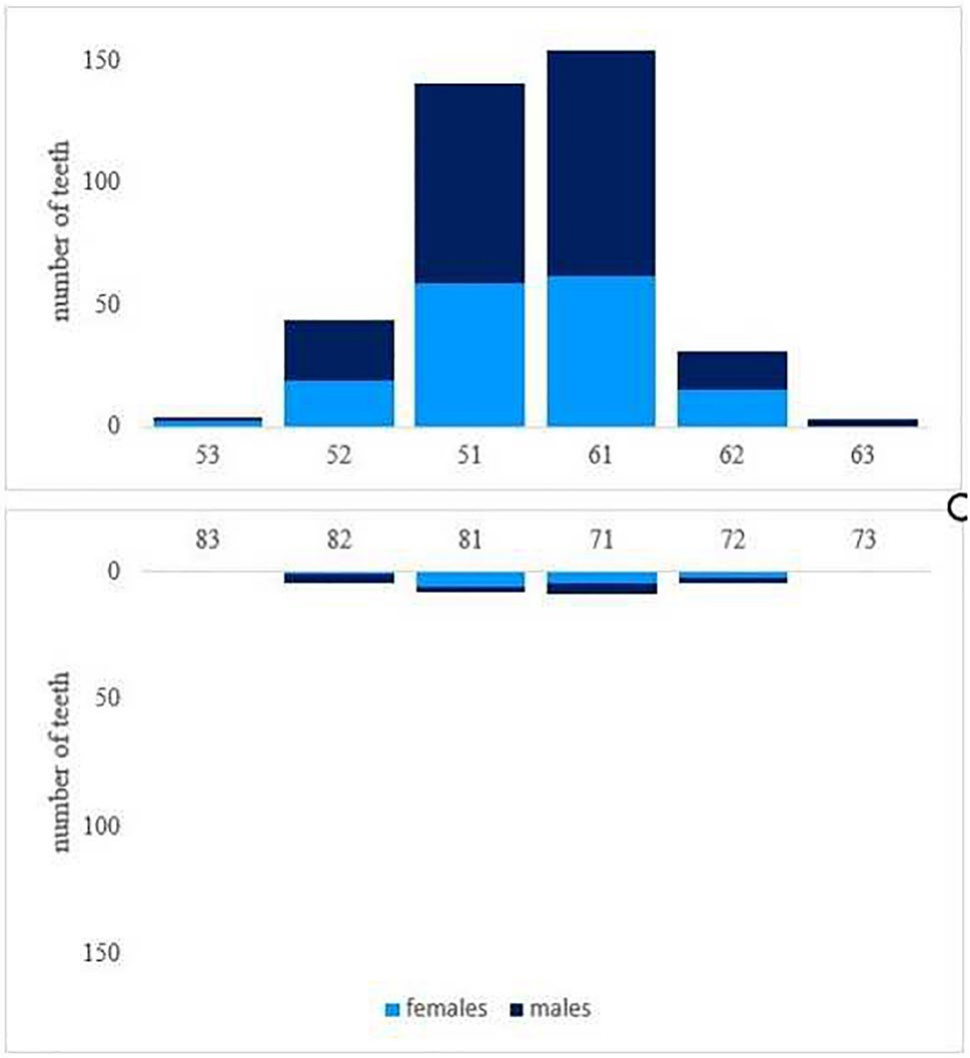
Distribution of traumatized teeth type per gender

Ninety-eight percent (*n* = 241) of cases presented with first-time dental trauma, whereas 2% (*n* = 5) had previously suffered from TDIs. Accompanying soft tissue injuries (laceration, abrasion, etc.) were present in 95 (39.1%) patients. In 52% (*n* = 127) of cases, only one deciduous tooth was affected. Twenty-nine percent (*n* = 72) showed two traumatized teeth within the same incident. The distribution of the number of affected teeth per traumatic incident is listed in Table 3 in relation to gender. Figure 4 shows the distribution of TDIs regarding tooth identification number and gender. Maxillary teeth were involved significantly more often (*n* = 229, 93.1%). Upper central incisors were found to be the most frequently affected by dental trauma (*n* = 296, 73.5%), followed by upper lateral incisors (*n* = 75, 18.6%). In 4.5% (*n* = 11) of cases, TDI occurred in teeth in the lower jaw and in 2.4% (*n* = 6) in the upper and lower jaw at the same time. In 97% (*n* = 391), a single injury type was diagnosed, the majority being dislocations (*n* = 298, 74%). The most common type of dislocations was subluxation (*n* = 85, 28.5%), followed by lateral dislocation (*n* = 61, 20.5%) and intrusion (*n* = 52, 17.4%). Forty-three patients suffered from avulsions (14.4%). Twenty-three percent (*n* = 92) of injuries were classified as fractures. In 3% (*n* = 12) of patients, both periodontal und hard dental tissue injuries were present with the most frequent combined injury being crown fractures along with increased tooth mobility (*n* = 4) The distribution of fractures, dislocations, and combined injuries is shown in Table 4. Table 5 presents an incidence matrix that gives thorough insight into how many times what type of TDI occurred. It also includes all possible variations of combined injury types. For example, 56 patients suffered from intrusions. In 54 out of these 56 intrusion cases, no other injury was observed. In 2 cases, however, the intrusion occurred in combination with crown fractures.

**Table 3:** Number of injured primary teeth per trauma and gender

	Number of injured primary teeth									
	1		2		3		4		5	
	*n*	%	*n*	%	*n*	%	*n*	%	*n*	%
All	127	52	72	29	31	13	13	5	3	1
Male	79	54	41	28	18	12	6	4	2	1
Female	48	48	31	31	13	13	7	7	1	1

**Table 4:** Distribution of injury type

Type of trauma	Classification	*n*	%
Fracture		92	23.0
	EF	1	1.1
	CF	48	52.2
	CFP	18	19.6
	CRF	19	20.7
	RF	3	3.3
Dislocation		298	74.0
	CCS	33	11.1
	LOS	85	28.5
	LDL	61	20.5
	INT	52	17.4
	EXT	21	7.0
	AVU	43	14.4
Combined injury		12	3.0
	EF	3	25.0
	CF	8	66.7
	CFP	1	8.3
	LOS	7	58.3
	LDL	3	25.0
	INT	2	16.7

**Table 5:** Incidence matrix for TDI

	**EF**	**CF**	**CFP**	**CRF**	**RF**	**CCS**	**LOS**	**LDL**	**INT**	**EXT**	**AVU**
EF	4	0	0	0	0	0	3	0	0	0	0
CF	0	56	0	0	0	0	4	2	2	0	0
CFP	0	0	19	0	0	0	0	1	0	0	0
CRF	0	0	0	19	0	0	0	0	0	0	0
RF	0	0	0	0	3	0	0	0	0	0	0
CCS	0	0	0	0	0	33	0	0	0	0	0
LOS	3	4	0	0	0	0	92	0	0	0	0
LDL	0	2	1	0	0	0	0	64	0	0	0
INT	0	2	0	0	0	0	0	0	54	0	0
EXT	0	0	0	0	0	0	0	0	0	24	0
AVU	0	0	0	0	0	0	0	0	0	0	43

In analyzing different causes of TDI, falling accidents proved to be the most prevalent reason in dental trauma in primary teeth (86%, *n* = 209; male: *n* = 124, 86.1%; female: *n* = 80, 85.9%). Only 6 males (4.2%) and 3 females (3.0%) suffered dental trauma through punching. In 3.5% of males (*n* = 5) and 3.0% of females (*n* = 3), dental trauma occurred due to collision accidents (Table 6). The correlation between accident and injury types established by crosstabulations shows that punching mostly caused dislocations (*n* = 15, 93.8%). The number of fractures and combined injuries increased due to falling or collision accidents. In 88.5% (*n* = 215) of cases, the traumatic incident occurred at home. TDIs due to accidents at preschools or schools were identified in 3.7% (*n* = 9) of cases. Accidents during leisure activities were observed in 2.5% (*n* = 6) and in traffic in 1.6% (*n* = 4).

**Table 6:** Accident and injury types of TDI

Accident type	Gender						Injury type					
	**Total**		**Male**		**Female**		**D**		**F**		**C**	
	***n***	**%**	***n***	**%**	***n***	**%**	***n***	**%**	***n***	**%**	***n***	**%**
**Punching**	9	3.7	6	4.2	3	3.0	15	93.8	1	6.2	0	0.0
**Falling**	209	86.0	124	86.1	85	85.9	260	73.9	80	22.7	3.1	0
**Collision accidents**	8	3.3	5	3.5	3	3.0	10	83.3	2	16.7	0	0.0
**Not known**	17	7.0	9	6.2	8	8.1	13	56.5	9	39.1	1	4.3

## Discussion

Traumatic dental injuries present a challenging health problem in many societies, especially in children. However, in the literature, only few reports address TDIs to the primary dentition. The risk for dental trauma in deciduous teeth is especially high between the ages of 2 and 3 years old. During this time period, motor coordination is developing and children of both genders are learning to walk [14]. Hence, the risk for falling accidents increases. The presented analysis of etiology of dental trauma showed that falling accidents were determined as the main cause of TDIs (male: *n* = 118, 86%; female: *n* = 80, 85%). With falling being the predominant cause of TDIs, the frequency of TDI occurrence on weekdays and on weekends is expected to be similar in young patients. The presented data confirms this assumption as the frequencies of TDI occurrence only show minimal variations in regard to the day of week. As other recent studies have shown similar results, the relative likelihood of a TDI occurrence being higher from May to November may be linked to the fact that outdoor activities entailing higher risks of falling accidents increase in spring and summer [18, 19]. Oldin et al. [20] showed that the frequencies of different causes for TDI vary with age. In older children and adolescents with permanent teeth, falling remains the dominant cause for TDIs. However, falling accidents are now primarily linked to sports and behavioral factors [20]. It was observed that boys are more prone to risky and aggressive behavior than girls. Yet, more recent studies have proven that the prevalence in male and female patients is equalizing in older children as girls are developing similar athletic interests and are exposed to the same risk factors in western countries as boys [3, 21]. In the presented study, the prevalence of dental trauma in deciduous teeth was significantly higher in boys compared to girls (m:f = 1:1.45, *p* = 0.048). This correlates with demographic evaluations of previous literature [18, 22]. Compared to gender ratio of TDIs in the permanent dentition, however, the difference between male and female patients is less prominent [14]. This difference may be attributed to varying etiologic factors within different age groups. Further, the relative share of TDIs in regard to gender shows no significant difference from the ages of 0 to 7 years old. From the ages of 8 to 10 years old, however, only boys were affected by TDIs in primary teeth. Multiple studies have confirmed that the eruption times of permanent teeth are earlier in females than males [23–25]. Hence, it is possible that the chance of TDIs in primary teeth at the ages of 8 to 10 years old is consequently lower in girls due to already erupting permanent teeth. In regard to the different accident types, boys prove to be the dominant gender in all categories. Interestingly, the gender gap narrows the more teeth are affected in one incident. TDIs affecting one, two, or three teeth occurred more often in boys. However, TDIs including four teeth were observed more often in girls than boys (m: *n* = 6, f: *n* = 7).

Most patients initially sought initial treatment at the Department of Emergency Dental Care at the University Dental Clinic of Vienna (*n* = 187, 77%) during clinic hours on weekdays. Only 11.4% (*n* = 28) presented as referrals. This confirms a rather high awareness level of the parents of the presented patient collective to consult specialized dental departments rather than common emergency departments in children’s hospitals for TDIs. Almost two-thirds of patients (*n* = 144, 65.2%) arrived within the first 24 h after TDI at the clinic. After 1 week, 89.1% of patients with TDIs (*n* = 197) had presented at the clinic. Reports have suggested that the most common reasons for considerable time lapses in some cases are underestimation of TDIs and its potential complications in the primary dentition. Prolonged transit time or parental unavailability also played into late arrival times [26, 27]. This emphasizes the importance of decentral dental care units within appropriate distance. As TDIs fairly evenly occur on weekdays as well as on weekends, opening hours of these support units should be extended to weekends.

Evaluating the pattern of affected teeth, the results show that upper central incisors were the most frequently injured teeth (73.5%). This is coherent with international data [1, 18, 28]. With 83.3%, De Amorim et al. observed a slightly higher value in a specialized pediatric practice in Brasil from 1993 to 2008 [29]. Due to their anterior position, upper incisors are more prone to be affected in traumatic incidents. Additional risk factors include insufficient lip closure, an overjet over 3 mm, and protrusion of upper anterior teeth [22, 30–32]. Overbite and canine classification have also been linked to higher incidence of TDI in deciduous teeth [22, 30, 33]. In contrast, lower incisors are more likely to be protected from TDI by lower lip and upper incisors. In this study in only 4.7%, TDIs occurred in lower teeth.

Based on the analysis of the presented data, dislocations were more prevalent than fractures or combined injuries. This confirms international trends of dislocations being the most common injury type in deciduous teeth due to the elasticity of alveolar bone and lower crown-root-relation at a younger age [14, 18]. With 74% of TDIs being dislocations, the percentage is slightly higher compared to the results of a recently published data report by Agouropoulos et al. who observed luxation injuries in 69% of cases in the primary dentition [18]. In the presented study, subluxations were the most frequent subtype of dislocation injuries (28.5%). This compared well to the results of De Amorim et al. (35.1%) [29]. Yet, the presented result is lower to previous findings of Mendoza-Mendoza et al. [34] in a retrospective cohort study on Spanish children between 0 and 7 years old (47.29%). At the same time, 17.4% intrusions and 14.4% avulsions compare well to Mendoza-Mendoza’s results in 2015 (intrusions: 23.15%, avulsions: 13.63%) [34]. Due to varying classification systems, however, the grounds for comparing incidence and prevalence in reference to different injury types and their subgroups with other notable studies are limited [35].

In addition, the findings of this study reveal that in 88.3% of cases the traumatic incident occurred at home. This is consistent with the results of a literature review that identified accidents within and around the home as the major source of TDI in deciduous teeth [14]. Other studies show that the main occurrence of TDIs is at home (43.5%), followed by traumatic incidents at school (10.1%) [29]. With 3.9%, the findings of this study, however, reveal a lower rate of TDI occurrence at school. Accidents during leisure activities including sports were observed in 2.6% of cases. Therefore, educational campaigns about the risk of dental trauma and prevention methods in order to increase health awareness and anticipatory guidance for families and teachers should be advised. Besides the previously mentioned oral predisposing factors, individual behavioral characteristics play an essential role in risk evaluation. To reduce the risk and incidence of dental trauma during sports, the use of protective athletic appliances such as mouth guards is considered an important preventative measure in older children and adolescents [36, 37]. Yet the motivation and compliance of younger children to wear such an appliance need to be further examined as they might present as a veritable limitation. Additionally, concerns in regard to their necessary regular renewal due to growth and development with age need to be considered in a thorough risk–benefit analysis before endorsing mouth guards for smaller children in clinical practice. The fact that only 1.3% (*n* = 3) of TDIs were caused in traffic can most likely be explained with the age groups represented in this study. A study has shown that dental trauma resulting from traffic accidents occurs most often in the second and third life decades [15].

This study’s findings should be framed by acknowledging its limitations. The analysis performed included a cohort of 243 patients that presented during opening hours on weekdays and on weekends at the Department of Emergency Dental Care at the University Dental Clinic in Vienna. Dental trauma that presented with local dentists or other emergency units was not taken into account. To gain a more comprehensive overview for Vienna, a multicenter approach including local dental offices is necessary. Furthermore, treatment outcomes were not evaluated in the presented study. It must also be acknowledged that due to varying classification systems the comparison of prevalence in reference to different injury types with other notable studies was difficult to assess. Combined injuries are often not examined as a separate entity. This underlines the importance of epidemiology and the study of patterns and causes of TDIs at a national and international population level on the basis of a standardized approach to reporting, classification, and methodology. Hence, a multicenter approach with the implantation of standardized classification protocols is recommended in order to gain a more comprehensive analysis of incidence, prevalence, and patterns of TDIs in deciduous teeth in Austria; develop national references; and explore regional differences. Standardized documentation in conjunction with the installation of prospective databases for initial assessment, treatment, and follow-up of treatment outcome is strongly recommended.

## Conclusion

The presented study is the first research on dental trauma in primary teeth in Austria. By large, the results correlate with previously published international data. Due to the high prevalence of TDIs in children, dental injuries present a frequent occurrence in daily practice worldwide. As their accurate treatment often proves to be a challenge to dentists in their daily practice, further education and training of dentists in the field of dental traumatology is strongly advised. The establishment of a regional network of trained dentists would be favorable. In this regard, online introductions such as the dental trauma guide can be useful tools. Furthermore, the city’s approach of decentralized care structures should be continued and the installation of more decentral support units and immediate dental care facilities within appropriate distance offering standardized documentation and treatment protocols should be encouraged.
